# Subcutaneous immunoglobulin replacement therapy in patients with immunodeficiencies – impact of drug packaging and administration method on patient reported outcomes

**DOI:** 10.1186/s12865-024-00608-0

**Published:** 2024-02-20

**Authors:** R. Mallick, G. Solomon, P. Bassett, X. Zhang, P. Patel, O. Lepeshkina

**Affiliations:** 1 CSL Behring, King of Prussia, PA USA; 2 Association des Patients Immunodeficients du Québec, Québec, Canada; 3 Meridian HealthComms Ltd, Manchester, UK; 4 Formerly of CSL Behring, King of Prussia, PA USA; 5 Centre hospitalier de l’Université Laval, Québec, Canada

**Keywords:** Immunodeficiency, Subcutaneous immunoglobulin (SCIg), Packaging method, Patient reported outcomes, Pre-filled syringes (PFS), Manual push, Pump, Treatment satisfaction questionnaire for medication (TSQM), Vials

## Abstract

**Background:**

Here, the perspective of patients with primary and secondary immunodeficiency receiving subcutaneous immunoglobulin (SCIg) via introductory smaller size pre-filled syringes (PFS) or vials were compared.

**Methods:**

An online survey was conducted in Canada by the Association des Patients Immunodéficients du Québec (APIQ) (10/2020–03/2021). Survey questions included: reasons for choosing SCIg packaging and administration methods, training experiences, infusion characteristics, and switching methods. The survey captured structured patient-reported outcomes: treatment satisfaction and its sub-domains, symptom state, general health perception, and physical and mental function. Respondents using PFS were compared with vial users, overall and stratified by their administration method (pump or manual push).

**Results:**

Of the 132 total respondents, 66 respondents used vials, with 38 using a pump and 28 using manual push. PFS (5 and 10 mL sizes) were being used by 120 respondents, with 38 using a pump and 82 using manual push. PFS users were associated with a 17% lower median (interquartile range) SCIg dose (10 [8, 12] vs. 12 [9, 16] g/week, respectively), a significantly shorter infusion preparation time (15 [10, 20] vs. 15 [10, 30] mins, respectively), and a trend for shorter length of infusion (60 [35, 90] vs. 70 [48, 90] mins, respectively) compared with those on vials. Patient-reported treatment satisfaction scores were overall similar between vial and PFS users (including on the domains of *effectiveness* and *convenience*), except for a higher score for vials over PFS on the domain of *global satisfaction* (*p*=0.02).

**Conclusions:**

Consistent with prescribing that reflects a recognition of less wastage, PFS users were associated with a significantly lower SCIg dose compared with vial users. PFS users were also associated with shorter pre-infusion times, reflecting simpler administration mechanics compared with vial users. Higher global satisfaction with treatment among vial users compared with PFS users was consistent with users being limited to smaller PFS size options in Canada during the study period. Patient experience on PFS is expected to improve with the introduction of larger PFS sizes. Overall, treatment satisfaction for SCIg remains consistently high with the introduction of PFS packaging compared with vials.

**Supplementary Information:**

The online version contains supplementary material available at 10.1186/s12865-024-00608-0.

## Background

Primary and secondary immunodeficiency diseases (PIDs and SIDs, respectively) are both disorders of the immune system that predispose individuals to an increased rate and severity of infections, and non-infectious complications including allergies, malignancies, autoimmune diseases, and lymphoproliferative and granulomatous manifestations [1–4]. PIDs refer to a heterogeneous group of genetic disorders characterized by an intrinsic impairment within the immune system [5, 6]. SIDs are caused by non-inherited factors that adversely affect the immune response [3, 7, 8]. The clinical manifestations of PID and SID usually include recurrent or complicated infections of the upper and/or lower respiratory tract, caused by encapsulated bacteria [7–9].

Patients with chronic diseases, such as PID, are vulnerable to significant disease burden that can negatively impact their physical function, emotional well-being, work productivity, social interactions, and family life [10–16]. Regular long-term treatment regimens can impose a treatment-related burden that interferes with daily life, increases the risk of adverse events, and acts as a constant reminder of the disease [10, 16–21]. Reduced treatment complexity and length of procedure has been shown to decrease treatment burden and have a positive impact on patient compliance and overall quality of life (QoL) [19].

Treatments for PID and SID include prophylactic antibiotic therapy, immunosuppressants to improve symptom control in cases of non-infectious conditions, and immunoglobulin replacement therapy (IgRT) [3, 9, 22]. Lifelong IgRT is the standard of care for patients with PID associated antibody deficiency, and is known to reduce infections, morbidity, and mortality [23, 24]. There is also growing evidence to support the use of IgRT in patients with SID [22, 25–28], as instances of SID can persists for ~2 years, and in some cases antibody levels never recover, and so require long-term IgRT [28]. IgRT can be administered either intravenously (IVIg) or subcutaneously (SCIg), with both routes of administration reported as effective and well-tolerated [20, 29, 30].

Injectable medications such as SCIg are traditionally packaged in vials and often require multiple stages of manual handling to prepare the medication for use [31]. The contents of the vial must first be transferred into a regular syringe using a transfer device, before fitting the syringe into an infusion pump for mechanical infusion [31]. For self-administration of SCIg, patients may be required to pool multiple vials or draw up SCIg into a large syringe before fitting it into a pump. These manual handling processes not only increase the risk of the medication becoming contaminated, they also require confidence and dexterity, which may prove difficult for some patients [31].

**Fig. 1:**
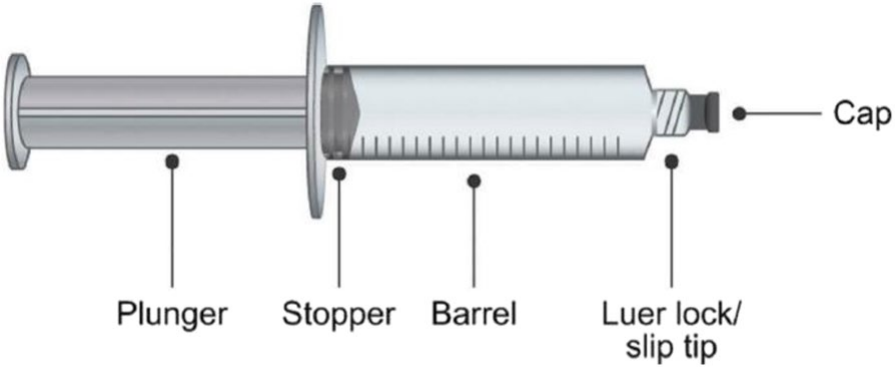
Design of PFS. Figure was repurposed from Ayman R. Kafal, Donald C. Vinh & Mélanie J. Langelier [31] Prefilled syringes for immunoglobulin G (IgG) replacement therapy: clinical experience from other disease settings, Expert Opinion on Drug Delivery, 15:12, 1199-1209, https://doi.org/10.1080/17425247.2018.1546692 and is covered by the Creative Commons Attribution-NonCommercial-NoDerivatives License. PFS, pre-filled syringes

Over the past decade, the drug delivery industry has developed novel drug packaging methods in an attempt to enhance patient convenience, experience, compliance, and outcomes [31, 32]. Pre-filled syringes (PFS) (Figure 1) were first developed for the administration of heparin and contain the injectable medication in a ready-to-use formula [32]. PFS have been reported to be effective and preferred over vials by patients and healthcare professionals alike [32–38] and could offer a simple and convenient alternative SCIg packaging method for use by patients with immunodeficiencies. Indeed, a recent study of the perspectives of patients with PID and SID receiving PFS SCIg highlighted several attributes contributing towards patient preference for PFS over other methods of IgRT delivery, including simplicity of administration, greater independence, greater convenience, and reduced treatment burden [39]. Other advantages of PFS include the provision of drugs in a sealed system, accurate dosing, reduced preparation time, reduced risk of contamination, and reduced drug wastage [32, 34].

SCIg has typically been administered via a mechanical pump. Depending on the patient, 5–50 mL can be infused per infusion site, usually over 2 hours [29, 40]. Historically, there has been somewhat limited compatibility between pumps and available PFS sizes, resulting in the need for a ‘tip-to-tip’ transfer step, where the SCIg is typically transferred from a smaller PFS to a larger regular syringe, compatible in turn with a typical 50- or 60-mL pump. Accordingly, early experience of patients exposed only to smaller 5 and 10 mL PFS sizes are potentially likely to reflect some of this inconvenience, and may conceivably only be resolved by the most recent introduction of a 20 and 50 mL PFS size [41, 42].

An alternative mode of SCIg administration is the manual push technique, which is performed using a syringe and butterfly needle to manually administer SCIg under the skin [43–45]. Manual push administration delivers SCIg over short intervals determined by the patient’s comfort level and has been shown to be a safe and effective treatment that is preferred over pumps by some patients with immunodeficiencies [43–45]. The manual push technique has the potential to further reduce treatment complexity, time commitment of procedure, and burden by eliminating the need to fit syringes into an infusion pump [31]. Indeed, previous studies of patients with immunodeficiencies suggest that QoL was improved after switching from an infusion pump to manual push due to increased freedom, flexibility, comfort, and not having to rely on an infusion pump [46, 47]. The fewer steps required for self-administration of SCIg with PFS and manual push could result in a reduction in the amount of training required, and greater ease of use for patients, factors that are likely to have a positive impact on adherence and patient satisfaction [35, 48]. Notably, treatment satisfaction of manual push with PFS may not be impacted by the additional inconvenience of tip-to-tip transfers from smaller PFS sizes to larger conventional syringes which may be needed in order to be compatible with pump administration. Multiple packaging and administration options for SCIg delivery allow treatment regimens to be tailored to suit an individual patient’s needs, circumstances, and lifestyle [20].

This study was motivated by findings from a recent study highlighting the impact of IgRT infusion methods on patient-reported outcomes (PROs) [30] and by the belief that characterization of early real-world experience of patients on smaller SCIg PFS sizes will yield insights that permit better evidence-based decision making, including potentially additional PFS offerings.

## Results

**Fig. 2:**
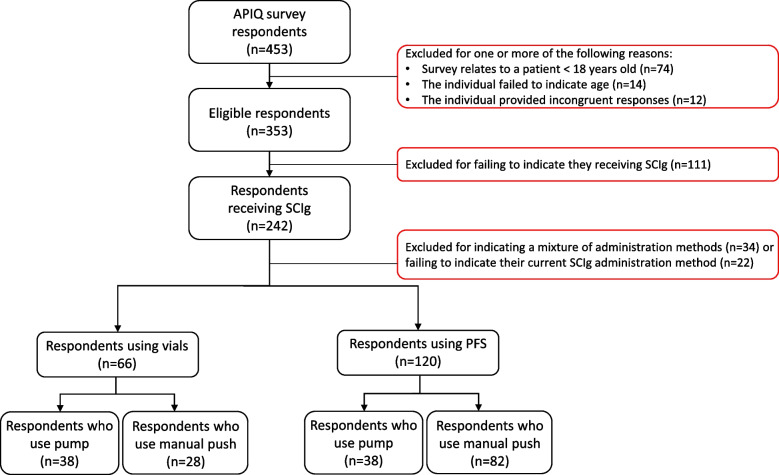
Criteria used to include respondents in the study. IgRT, immunoglobulin replacement therapy; PFS, pre-filled syringes; SCIg, subcutaneous immunoglobulin

Of the 453 respondents to an Association des Patients Immunodéficients du Québec (APIQ) survey [30], 74.0% (*n*=242) indicated SCIg as their current IgRT infusion method (Figure 2). SCIg respondents who indicated they used a mixture of SCIg packaging methods (*n*=34) or who failed to indicate their current SCIg packaging or SCIg administration method (*n*=22) were excluded, leaving 186 respondents in our analysis. Overall, 120 (64.5%) respondents indicated PFS and 66 (35.5%) respondents indicated vials as their current SCIg packaging method. Of the 186 respondents, 76 (40.9%) indicated pump as their chosen SCIg administration method, with 38 (50%) of these using PFS and an equal 38 (50%) using vials. The remaining 110 (59.1%) respondents indicated manual push as their chosen SCIg administration method, with 82 (74.5%) using PFS and 28 (25.5%) of these using vials. No significant differences were found in respondent characteristics between the PFS and vial cohorts overall, including patient weight (Table 1), or in the pump and manual push administration method subgroups (Additional files 1 and file 2).

**Table 1:** Summary of respondent characteristics of the vial and PFS cohorts

**Respondent characteristics (*n*=220)**		**Vial cohort (A)**		**PFS cohort (B)**		***p* values**
		**Summary**	**n**	**Summary**	**n**	**A vs. B**
Age (years), median [IQR]		57 [48, 66]	66	59 [49, 66]	120	0.34
Age at diagnosis (years), median [IQR]		46 [25, 59]	65	42 [25, 42]	118	0.99
Gender, n (%)	Female	39 (60.0%)	65	76 (63.9%)	119	0.61
	Male	26 (40.0%)		43 (36.1%)		
Bodyweight (kg), mean (± SD)		75.3 (± 17.2)	58	76.0 (± 17.5)	109	0.79
Underlying condition, n (%)	CVID	25 (47.2%)	53	37 (40.2%)	92	0.48
	IgG Sub	13 (24.5%)		26 (28.3%)		
	DGS	2 (3.8%)		4 (4.4%)		
	SID	9 (17.0%)		10 (10.9%)		
	Other^a^	4 (7.6%)		15 (16.3%)		
Years since diagnosis, n (%)	< 2 years	4 (6.2%)	65	10 (8.5%)	118	0.22
	2–9 years	34 (52.3%)		46 (39.0%)		
	≥ 10 years	27 (41.5%)		62 (52.5%)		
Time on IgG, n (%)	< 1 year	3 (4.6%)	66	5 (4.2%)	120	0.56
	1–2 years	67 (10.6%)		21 (17.5%)		
	2–3 years	4 (6.1%)		17 (14.2%)		
	4–6 years	21 (31.8%)		18 (15.0%)		
	≥ 6 years	31 (47.0%)		59 (49.2%)		
Current treatment experience, n (%)	< 2 years	8 (12.1%)	66	21 (17.8%)	118	0.26
	2–9 years	44 (66.7%)		64 (54.2%)		
	≥ 10 years	14 (21.2%)		33 (28.0%)		
Antibiotics before IgG, n (%)	No	44 (77.2%)	57	79 (74.5%)	106	0.71
	Yes	13 (22.8%)		27 (25.5%)		
Antibiotics since starting IgG, n (%)	No	24 (36.9%)	65	50 (43.9%)	114	0.37
	Yes	41 (63.1%)		64 (56.1%)		

Categorical variables were analyzed using chi-squared tests, and continuous variables were analyzed using Analysis of Variance (ANOVA) post-hoc tests if found to be normally distributed, or Mann Whitney tests if otherwise. Significant p-values are highlighted in bold

^a^Other indications are: X-linked agammaglobulinemia (vial, *n*=3; PFS, *n*=3), severe combined immunodeficiency (vial, *n*=0; PFS, *n*=4), specific antibody deficiency (vial, *n*=1; PFS, *n*=3), idiopathic autoimmune hemolytic Anemia (vial, *n*=1; PFS, *n*=0), autoimmune disease (vial, *n*=1; PFS, *n*=1), hypogammaglobulinemia (vial, *n*=1; PFS, *n*=1), chronic lymphocytic leukemia (vial, *n*=1; PFS, *n*=0), Waldenstrom macroglobulinemia (vial, *n*=1; PFS, *n*=0). CVID, common variable immune deficiency; DGS, DiGeorge syndrome; GHP, general health perception; GMH-2, global mental health 2; GPH-2, global physical health 2; IgG, immunoglobulin; IgG Sub, immunoglobulin subclass deficiency; IQR, interquartile range; kg, kilogram; PFS, pre-filled syringes; SCIg, subcutaneous immunoglobulin; SD, standard deviation; SID, secondary immunodeficiencies

### Choosing a SCIg packaging method

**Fig. 3:**
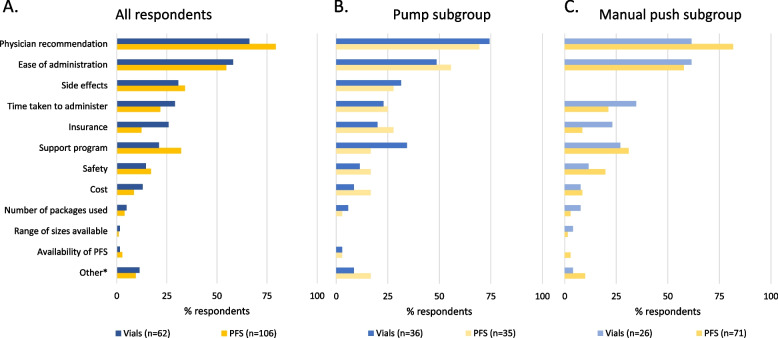
Clustered bar chart of respondents’ reasons for choosing a SCIg packaging method, stratified by SCIg administration method (pump and manual push). (**A**) All respondents, (**B**) pump subgroup, and (**C**) manual push subgroup. PFS, pre-filled syringes; SCIg, subcutaneous immunoglobulin

To understand the factors taken into consideration by patients when choosing a SCIg packaging method, information on the most important reasons a method was chosen was collected for the two packaging cohorts (Figure 3A, B and C). A physician’s recommendation was the most common factor for respondents in both the PFS and vial cohorts (79.3% [*n*=84] and 66.1% [*n*=41]) respectively; Figure 3A). Respondents in the PFS cohort were significantly less likely to state insurance as an important factor compared with respondents in the vial cohort (12.3% [*n*=13] vs 25.8% [*n*=16], *p*=0.03, Figure 3A).

When asked how their QoL would change if they could have their medication delivered to their home, the vast majority of respondents in the PFS and vial packaging cohorts stated that their QoL would improve or substantially improve (83.3% [*n*=15] and 72.8% [*n*=8], respectively). Similar observations were seen in the SCIg packaging cohorts in the pump subgroup, with no statistical difference between the two cohorts (*p*>0.05 for all comparisons). Insufficient data was available for the manual push subgroup to perform similar analyzes.

### Self-infusion training experience

To understand the impact of SCIg packaging and SCIg administration methods on self-infusion training experience and satisfaction, information regarding training characteristics was collected (Table 2, Additional files 3 and 4). An interaction between SCIg packaging and SCIg administration methods was identified for the number (*p*=0.002) and length (*p*=0.06) of training sessions, suggesting that the effect of SCIg packaging on these characteristics varied depending on the SCIg administration method. In the pump subgroup, both the number and length of training sessions were significantly higher for the PFS cohort compared with the vial cohort (*p*=0.007 and *p*=0.004, respectively; Table 2). Conversely, in the manual push subgroup, the number of training sessions was lower in the PFS cohort compared with the vial cohort, although this result did not quite reach statistical significance (*p*=0.07, Additional file 4).

**Table 2:** Self-infusion training experiences of the vial and PFS cohorts in the pump subgroup

**Training characteristics (Pump)**		**Vial cohort (A)**		**PFS cohort (B)**		***p* values**
		**Summary**	**n**	**Summary**	**n**	**A vs. B**
Number of training sessions, n (%)	1	21 (58.3%)	36	11 (30.6%)	36	**0.007**
	2	10 (27.8%)		13 (36.1%)		
	3	5 (13.9%)		6 (16.7%)		
	4	0 (0.0%)		3 (8.3%)		
	5	0 (0.0%)		0 (0.0%)		
	>5	0 (0.0%)		3 (8.3%)		
Location of training, n (%)	Doctors	1 (2.8%)	36	2 (5.6%)	36	0.57
	Home	16 (44.4%)		19 (52.8%)		
	Hospital	16 (44.4%)		10 (27.8%)		
	Inf Center	2 (5.6%)		2 (5.6%)		
	Other^a^	1 (2.8%)		3 (8.3%)		
Length of training session (hours)		1.3 [1.0, 2.0]	34	2.0 [1.5, 3.0]	34	**0.004**
Ease of learning to infuse, n (%)	Very difficult	2 (5.6%)	36	1 (2.8%)	36	0.98
	Difficult	3 (8.3%)		4 (11.1%)		
	Neither	5 (13.9%)		4 (11.1%)		
	Easy	12 (33.3%)		14 (38.9%)		
	Very easy	14 (38.9%)		13 (36.1%)		
Satisfaction with training, n (%)	Very dissatisfied	0 (0.0%)	36	0 (0.0%)	36	0.26
	Dissatisfied	0 (0.0%)		1 (2.8%)		
	Neither	0 (0.0%)		1 (2.8%)		
	Satisfied	9 (25.0%)		11 (30.6%)		
	Very satisfied	27 (75.0%)		23 (63.9%)		
Concerns during training, n (%)	Drawing drug	8 (23.5%)	34	3 (8.3%)	36	0.06
	Inserting needle	12 (35.3%)		18 (50.0%)		
	Using pump	3 (8.8%)		0 (0.0%)		
	Prime tube	1 (2.9%)		5 (13.9%)		
	Other^b^	0 (0.0%)		0 (0.0%)		
	No concerns	10 (29.4%)		10 (27.8%)		

Data were compared using Mann-Whitney test (ease of SCIg training, number of SCIg training sessions) or Fisher’s exact test (SCIg training location, type of SCIg trainer). Significant p values are in bold

*PFS* pre-filled syringes, *SCIg* subcutaneous immunoglobulin

^a^Other training locations: Institut de recherches cliniques de Montréal (vials, *n*=1; PFS, *n*=3) or a local community service center (vials, *n*=0; PFS, *n*=1)

^b^Other concerns: applying needles to tubing (vials, *n*=0; PFS, *n*=1)

When analyzing who provided training, a hospital nurse was found to be the most common provider in both cohorts (vial: 56.5% [*n*=35], and PFS: 61.0% [*n*=64]). The trainer was graded as ‘knowledgeable’ or ‘very knowledgeable’ by over 95.0% of respondents in both cohorts. The majority of respondents (PFS: 80.4% [*n*=86], and vials: 93.5% [*n*=57]) felt they were ‘confident’ or ‘very confident’ in self-administering SCIg following training. Overall, over 95.0% of respondents in all SCIg packaging cohorts were satisfied with their training, the majority of which were very satisfied (Table 2, Additional files 3 and 4). When asked what their greatest training concern was, the most commonly reported answer was inserting the needle (Table 2, Additional files 3 and 4).

### Self-infusion characteristics

#### SCIg dosing

PFS users were associated with a significantly lower median (interquartile range [IQR]) SCIg dose compared with vial users (10 [8, 12] vs. 12 [9, 16] g/week, respectively; *p*=0.02; Table 3) for both SCIg administration methods. Further, despite PFS and vial users having a similar average weight (Table 1), the PFS cohort was associated with a lower median (IQR) SCIg dose adjusted by patient bodyweight compared with the vial cohort (0.13 [0.10, 0.17] vs. 0.15 [0.11, 0.19] g/week/kg, respectively; Table 3), albeit with borderline significance (*p*=0.06, Table 3).

**Table 3:** SCIg dosing of the vial and PFS cohorts overall, and stratified by SCIg administration method (pump and manual push).

**Dosing**	**Vial cohort (A)**		**PFS cohort (B)**		***p* values**
	**Summary**	**n**	**Summary**	**n**	**A vs. B**
**All respondents**					
SCIg dose (g/week) (median [IQR])	12 [9, 16]	63	10 [8, 12]	110	**0.02**
SCIg dose per bodyweight (g/week/kg) (median [IQR])	0.15 [0.11, 0.19]	56	0.13 [0.10, 0.17]	100	0.06
**Pump subgroup**					
SCIg dose (g/week) (median [IQR])	12 [10, 14]	37	10 [8, 16]	37	0.30
SCIg dose per bodyweight (g/week/kg) (median [IQR])	0.15 [0.12, 0.19]	32	0.15 [0.11, 0.20]	34	0.66
**Manual push subgroup**					
SCIg dose (g/week) (median [IQR])	10 [8, 16]	26	10 [8, 12]	73	0.18
SCIg dose per bodyweight (g/week/kg) (median [IQR])	0.14 [0.11, 0.30]	24	0.12 [0.10, 0.16]	66	0.14

Data were compared using the Mann-Whitney test due to the non-normality (skewness) of the distributions. Significant *p*-values are highlighted in bold. g grams, *IQR* interquartile range, *kg* kilogram, *PFS* pre-filled syringes, *SCIg* subcutaneous immunoglobulin

#### SCIg infusion efficiency

To assess the burden of infusion treatment, information on the length of infusion steps and the number of infusion sites were collected (Table 4). The mean duration of infusion preparation time was significantly shorter in the PFS cohort compared with the vial cohort for both SCIg administration methods (16.7 minutes vs. 19.2 minutes, *p*=0.02, Table 4), although the median time was the same in both cohorts (15 minutes, Table 4). The length of infusions was numerically lower in both SCIg packaging cohorts in the manual push subgroup compared with the pump subgroup (vials: 60 minutes, and PFS: 50 minutes, vs. vials: 75 minutes, and PFS: 90 minutes; respectively; Table 4).

**Table 4:** SCIg infusion efficiency of the vial and PFS, cohorts, stratified by SCIg administration method (pump and manual push)

**Infusion characteristics**		**Vial cohort (A)**		**PFS cohort (B)**		***p* values**
		**Summary**	**n**	**Summary**	**n**	**A vs. B**
**All respondents**						
Infusion preparation time (mins) (median [IQR])		15 [10, 30]	64	15 [10, 20]	110	0.02^*^
Length of infusion (mins) (median [IQR])		70 [48, 90]	64	60 [35, 90]	110	0.07
Post-infusion clean up time (mins) (median [IQR])		5 [5, 10]	60	5 [5, 10]	108	0.89
Number of infusion sites, n (%)	1	2 (3.3%)	61	6 (5.7%)	106	0.12
	2 or 3	41 (67.2%)		83 (78.3%)		
	≥4	18 (29.5%)		17 (16.0%)		
**Pump subgroup**						
Infusion preparation time (mins) (median [IQR])		20 [15, 30]	37	15 [10, 20]	37	0.20
Length of infusion (mins) (median [IQR])		75 [60, 90]	37	90 [60, 105]	37	0.33
Post-infusion clean up time (mins) (median [IQR])		6 [5, 10]	34	9 [5, 15]	36	0.54
Number of infusion sites, n (%)	1	0 (0.0%)	36	2 (5.6%)	36	0.15
	2 or 3	21 (58.3%)		25 (69.4%)		
	≥4	15 (41.7%)		9 (25.0%)		
**Manual push subgroup**						
Infusion preparation time (mins) (median [IQR])		15 [10, 20]	27	10 [8, 15]	73	0.23
Length of infusion (mins) (median [IQR])		60 [40, 90]	27	50 [30, 75]	73	0.06
Post-infusion clean up time (mins) (median [IQR])		5 [2, 5]	26	5 [3, 10]	72	0.46
Number of infusion sites, n (%)	1	2 (8.0%)	25	4 (5.7%)	70	0.92
	2 or 3	20 (80.0%)		58 (82.9%)		
	≥4	3 (12.0%)		8 (11.4%)		

Data were compared using the Mann-Whitney test due to the non-normality (skewness) of the distributions. Significant *p*-values are highlighted in bold

*Although the median infusion preparation times are numerically similar for the vial and PFS cohorts, the difference between the two distributions is significant and is demonstrated by the mean (± standard deviation) values of 19.2 (± 12.4) mins and 16.7 (± 15.6) mins, respectively. *IQR* interquartile range, *mins* minutes, *PFS* pre-filled syringes, *SCIg* subcutaneous immunoglobulin

When asked how important respondents perceived the duration of infusions, nearly all respondents reported it as ‘important’ or ‘very important’ in their response, except for one (11.1%) PFS respondent in the manual push subgroup, who reported their perception as neither ‘important’ or ‘unimportant’.

### Patient-reported treatment satisfaction (TSQM-9)

#### TSQM-9 *effectiveness*

**Fig. 4:**
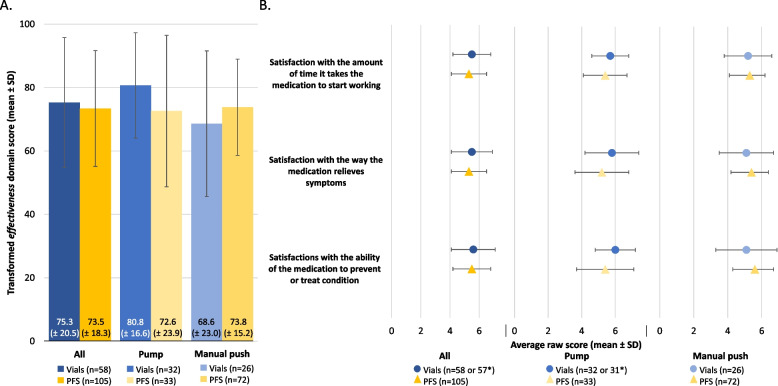
Perceived treatment *effectivenes*s in the vial and PFS cohorts, stratified by SCIg administration method (pump and manual push). (**A**) Transformed TSQM *effectiveness* domain scores and (**B**) raw scores from the corresponding TSQM domain items. *n numbers vary due to missing respondent data for various survey questions. Transformed domain scores are on a 0–100 scale from worst to best and the raw scores are on a 1 to 5 or 7 scale from extremely dissatisfied to extremely satisfied. Continuous variables were compared using an unpaired t-test and only the significant p values are included in the figure for brevity. PFS, pre-filled syringes; SCIg, subcutaneous immunoglobulin; SD, standard deviation; TSQM, treatment satisfaction questionnaire for medication

Analysis revealed a statistically significant interaction between SCIg packaging and SCIg administration methods for the Treatment Satisfaction Medication Questionnaire (TSQM-9) *effectiveness* domain scores, suggesting that the effect of SCIg packaging on TSQM-9 *effectiveness* varied depending on the SCIg administration method (*p*=0.04). However, there were only numerical score differences between the TSQM-9 *effectiveness* domain for the PFS and vial cohorts in the pump subgroup (80.7 vs. 72.6, respectively, *p*=0.11; Figure 4A), and manual push subgroup (68.6 vs. 73.8, respectively, *p*=0.20; Figure 4A). Similarly, when analyzing item specific evidence on the TSQM-9 *effectiveness* domain, although a statistical interaction between SCIg packaging and SCIg administration methods was identified for satisfaction *with the ability of the medication to prevent or treat your condition* (*p*=0.03) and *the way the medication relieves symptoms* (*p*=0.05); differences between the PFS cohort and the vial cohort were only numerical and not statistically significant in both SCIg administration subgroups (Figure 4B).

#### TSQM-9 *convenience*

**Fig. 5:**
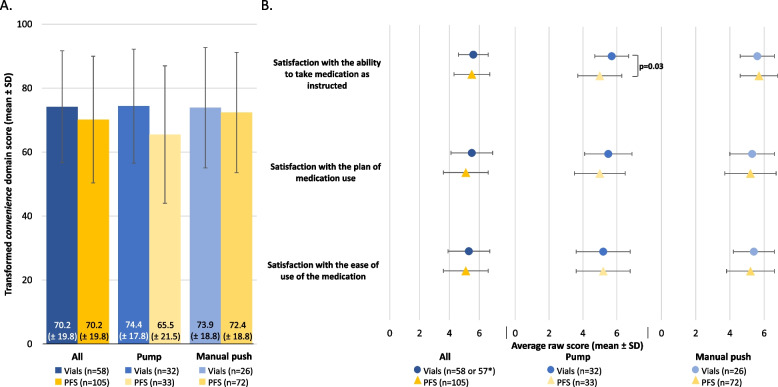
Perceived treatment *convenience* in the vial and PFS cohorts, stratified by SCIg administration method (pump and manual push). (**A**) Transformed TSQM *convenience* domain scores and (**B**) raw scores from the corresponding TSQM domain items. *n numbers vary due to missing respondent data for various survey questions. Transformed domain scores are on a 0–100 scale from worst to best and the raw scores are on a 1 to 5 or 7 scale from extremely dissatisfied to extremely satisfied. Continuous variables were compared using an unpaired t-test and only the significant *p* values are included in the figure for brevity. PFS, pre-filled syringes; SCIg, subcutaneous immunoglobulin; SD, standard deviation; TSQM, treatment satisfaction questionnaire for medication

There was no evidence of an interaction between SCIg packaging and SCIg administration methods for the TSQM-9 *convenience* domain scores (*p*=0.25), with no significant difference in scores between the vial and the PFS cohorts (*p*=0.2, Figure 5A). When analyzing item specific evidence on the TSQM-9 *convenience* domain, an interaction between SCIg packaging and SCIg administration methods was identified for satisfaction *with the convenience of taking the medication as instructed* (*p*=0.04). For this item, the PFS cohort scored lower than the vial cohort in the pump subgroup (5.0 vs. 5.7, respectively, *p*=0.03; Figure 5B), whereas there was no difference between the PFS and vial cohorts in the manual push subgroup (5.7 vs. 5.6, respectively, *p*=0.69; Figure 5B).

#### TSQM-9 *global satisfaction*

**Fig. 6:**
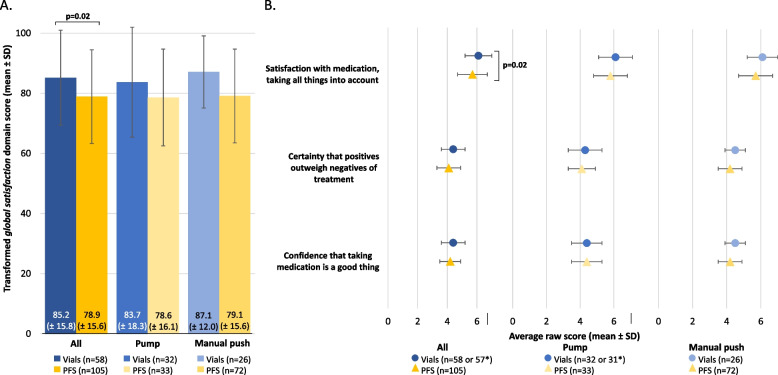
Perceived treatment *global satisfaction* in the vial and PFS cohorts, stratified by SCIg administration method (pump and manual push). (**A**) Transformed TSQM *global satisfaction* domain scores and (**B**) raw scores from the corresponding TSQM domain items. *n numbers vary due to missing respondent data for various survey questions. Transformed domain scores are on a 0–100 scale from worst to best and the raw scores are on a 1 to 5 or 7 scale from extremely dissatisfied to extremely satisfied. Continuous variables were compared using an unpaired t-test and only the significant p values are included in the figure for brevity. PFS, pre-filled syringes; SCIg, subcutaneous immunoglobulin; SD, standard deviation; TSQM, treatment satisfaction questionnaire for medication

There was no evidence of an interaction between SCIg packaging and SCIg administration methods for the TSQM-9 *global satisfaction* domain scores (*p*=0.58). The PFS cohort scored significantly lower compared with the vial cohort overall (78.9 vs. 85.2, respectively, *p*=0.01; Figure 6A). Similarly, when analyzing item specific evidence on the TSQM-9 *global satisfaction* domain, the PFS cohort scored significantly lower compared with the vial cohort for *satisfaction with medication taking all things into account* (5.7 vs. 6.1, respectively, *p*=0.02; Figure 6B).

### Patient-reported outcomes

**Fig. 7:**
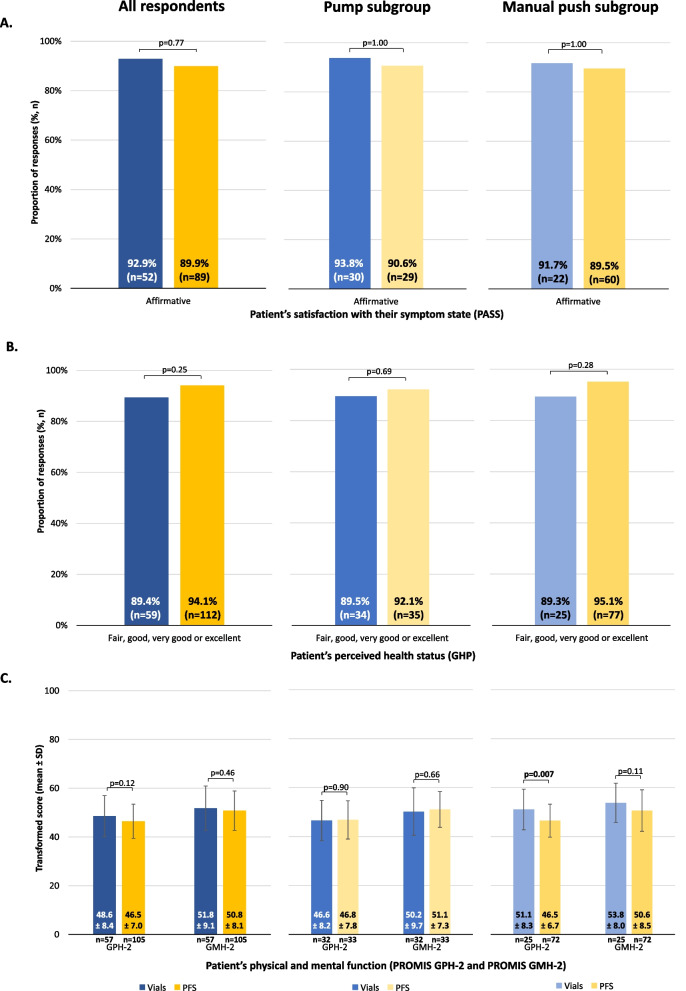
Respondent symptom state and perceived health status in the vial and PFS cohorts, stratified by SCIg administration method (pump and manual push). (**A**) Proportion of respondents who responded ‘affirmative’ to whether they were at an acceptable symptom state (as measured using PASS), (**B**) proportion of respondents who described their current health status as ‘fair’, ‘good’, ‘very good’ or ‘excellent’ (as measured using GHP), and (**C**) transformed scores for PROMIS GPH-2 and GMH-2. The complementary response category for PASS was ‘negative’ and for GHP were ‘poor’ or ‘very poor’. GHP, general health perception; GMH-2, global mental health 2; GPH-2, global physical health 2; IVIg, intravenous immunoglobulin; PASS, patient acceptable symptom state; PFS, pre-filled syringes; PROMIS, patient-reported outcomes measurement information system; SCIg, subcutaneous immunoglobulin

There were no significant interactions between SCIg packaging and SCIg administration methods on Patient Acceptable Symptom State (PASS), General Health Perception (GHP), and Patient Reported Outcomes Measurement Information System (PROMIS) Global Mental Health (GMH-2) measures, with no significant difference in scores between the PFS and the vial cohorts (all *p*>0.05, Figure 7). There was evidence of a significant interaction between SCIg packaging and SCIg administration methods for the PROMIS Global Physical Health (GPH-2) (*p*=0.05) suggesting that the effect of SCIg packaging on PROMIS GPH-2 varied depending on SCIg administration method. Respondents using PFS with manual push scored significantly lower for PROMIS GPH-2 compared with respondents using vials with manual push (46.5 vs. 51.1, respectively, *p*=0.008; Figure 7).

### Switching between SCIg packaging methods

Of the 186 respondents currently receiving SCIg, 51 had switched to their current SCIg packaging method from a previously used, different packaging method. Of these, 35 (68.6%) respondents switched to PFS packaging and 16 (31.4%) respondents switched to vial packaging. A physician’s recommendation was the most common reason given by respondents who switched to PFS (44.4% [*n*=8], whereas easier administration was the most common reason given by respondents in the vial cohort (63.6% [*n*=7]). Most respondents in both cohorts reported improved or substantially improved QoL following their switch (PFS: 66.6% [*n*=16] and vials: 75.0% [*n*=9]). Similarly, most of the respondents in both cohorts reported improved or substantially improved treatment satisfaction following their switch (83.4% [*n*=20] of the PFS cohort and (75.0% [*n*=9] of the vial cohort). The positive impact of switching SCIg packaging methods on mental health was significantly greater in the PFS cohort compared with the vial cohort (*p*=0.03), with 31.8% (*n*=7) of respondents who switched to PFS reporting improved mental health following switch, compared with 0.0% (*n*=0) of the vial cohort. Positive impacts on productivity, treatment compliance, and physical health were also observed for both cohorts after switching SCIg packaging methods, but to a lesser extent than those reported above, with 54.2% (*n*=13) of the PFS cohort and 58.3% (*n*=7) of the vial cohort, reporting an improved or substantially improved impact on productivity. In addition, 45.8% (*n*=11) of the PFS cohort and 33.3% (*n*=4) of the vial cohort reported an improved or substantially improved impact on treatment compliance. Meanwhile, 45.9% (*n*=11) of the PFS cohort and 41.6% (*n*=5) of the vial cohort reported an improved or substantially improved impact on physical health. No differences were observed between current and previous training experiences in the two SCIg packaging cohorts in any characteristics measured. There were only eight respondents who reported switching SCIg administration method; all of these respondents switched from pump to manual push administration.

## Discussion

In this analysis of a survey of Canadian respondents with PID or SID, we evaluated reasons for choosing SCIg packaging and administration methods, SCIg training experience, and self-infusion characteristics (including dosing and infusion efficiency, such as infusion preparation time, length of infusion, and post-infusion clean up time). We analyzed the impact of different SCIg packaging methods (suitably stratified by pump vs. manual push administration as needed) on patient-reported treatment satisfaction in terms of its various domains (perceived *effectiveness*, *convenience*, and *global satisfaction*), as well as in terms of underlying items (questions). In addition, patient-reported symptom status, overall well-being, and physical and mental health were investigated. Finally, details of switching between SCIg packaging methods were assessed.

In this survey, we found that the majority of respondents, regardless of SCIg administration method, had started to use newly available small (5 and 10 mL) PFS, with fewer respondents using vials. In fact, a larger proportion of patients had switched from vial packaging to PFS packaging compared with vice versa, and did so predominantly due to physician recommendation. One potential reason for the relatively high numbers of PFS users could be due to healthcare providers recommending it for patients with less motor skill-related functionality. This is consistent with evidence in this study that suggests poorer physical function in the PFS cohort compared with the vial cohort, specifically in those using the manual push administration method. These patients could potentially benefit from the reduced number of handling steps involved in the infusion process with PFS compared with vials and conventional syringes [31]*.* Parenthetically, this could potentially add an element of selection bias against the PFS cohort in the survey.

Engaging patients in the treatment decision process can empower patients, which can have positive impacts on treatment satisfaction and adherence [49, 50]. Consistent with previous reports [49], our findings have demonstrated that while patients value the ability to choose between treatment options, they also acknowledge their inability to make a completely informed choice due to their lack of clinical knowledge, and patient decision-making was largely influenced by the clinician or prescriber. Other factors that may influence choice of SCIg packaging could vary between the different cohorts due to the specific requirements of patients within each group [19, 35, 48].

The availability of SCIg packaging options at the time of treatment initiation could also influence patient choice. Indeed, from the authors experience, certain centers in Canada have trained patients exclusively with PFS since 2020. This is reflected in the survey data, with a higher proportion of patients in the PFS cohort having fewer than two years’ experience on their current treatment, as well as fewer than two years on IgRT overall, compared with the vial cohort. In addition, it is possible that health insurance policies could also influence patient choice due to the costs associated with the different SCIg packaging and SCIg administration methods, limiting the choice of some patients.

An important finding in this study is that patients in the PFS cohort, on average, received a significantly lower SCIg dose (approximately 17% less) compared with patients in the vial cohort. This evidence is consistent with the notion that medicinal products drawn via a transfer needle from vials into conventional syringes are associated with greater amounts of wastage compared with medicinal products packaged in PFS [32, 34, 51]. SCIg wastage results in the unnecessary depletion of immunoglobulin reserves and has cost implications for payers. Indeed, studies of other therapies have shown significant cost savings to payers following a transition from vials to PFS [31]. A recent study showed that there was a 6% reduction in hemodialysis costs following a switch from epoetin alfa vials to epoetin alfa PFS due to less drug waste with PFS compared with vials [52]. In addition, a 61% cost reduction was reported for the use of thiopental PFS compared with thiopental vials (£780 vs. £2036/year, respectively) in one UK hospital [53], and 16% cost savings were reported for ephedrine PFS compared with ephedrine vials for obstetrical anaesthesia (€2.6 vs. €3.1/patient, respectively, over a two-week period) [54]. Finally, a budget impact analysis by Benhamou et al revealed that despite use of more costly atropine PFS in France, there was a potential annual budget saving of about 37% compared with atropine vials (approx. €9 million vs. €14.3 million, respectively), attributed in part to a reduction in drug wastage [55].

While the survey was not designed to distinguish between the drawn dose and the dose received, the difference in dose between the PFS and vial cohorts (17%) could potentially be accounted for by knowledge of potential wastage with vials by prescribers and patients [31, 52–55]. From a prescriber perspective, given the need for rounding dose to accommodate minimum discrete 5 mL increments in SCIg volume, health care providers have traditionally rounded up (rather than rounded down) vial doses in real world practice to minimize perceived waste and loss of therapeutic effectiveness [56]. Yet, given a recent Canadian directive recommending greater scrutiny of Ig dosing, the coincident availability of PFS may have provided an opportunity for providers to round down dosing, including to ideal rather than actual patient weight. Additionally, the authors believe a transition to PFS may have been an opportunity to prescribe a dose based on ideal body weight rather than actual body weight. It is conceivable, however, that to some degree, fewer of the patients on higher doses may have been prescribed PFS packaging due to the limited maximal dose that can be delivered by the small 5 and 10 mL PFS that were available in Canada at the time this survey was conducted [57, 58].

In this study, the length of infusion preparation time was significantly shorter in the PFS cohort compared with the vial cohort. Other things equal, reduced infusion times are in theory likely to reduce the treatment burden perceived by patients, due to less disruption to daily life. Indeed, a recent study demonstrated that enhanced infusion efficiency was associated with substantially enhanced treatment satisfaction [59].

Patient reported treatment satisfaction for *both* PFS and vial packaging overall was consistent with the relatively high levels seen in numerous past studies of patients treated with SCIg [30, 59–62]. Treatment satisfaction was similar between the PFS and vial cohorts in terms of the domains of perceived *effectiveness* and *convenience* in the TSQM tool*.* However, the PFS cohort was seen to be associated with a significantly lower score on the TSQM domain of *global satisfaction* compared with the vial cohort overall. Several factors may have accounted for these findings. First, given previous evidence that longer duration of SCIg experience contributes to better treatment satisfaction [47], the lower *global satisfaction* scores associated with the PFS cohort may in part be due to a larger proportion of the PFS cohort having a more limited observed experience with their current SCIg packaging method, with IgRT overall, and conceivably also with SCIg self-infusion, compared with the vial cohort.

Second, PFS users may be less satisfied compared with vial users due to the incompatibility of small PFS sizes with typically larger pumps. Only 5 and 10 mL PFS sizes were available in Canada at the time of the survey [57, 58], meaning some patients may have needed to perform multiple tip-to-tip transfers from a PFS to a larger regular syringe compatible with a typical 50 or 60 mL pump. The sheer magnitude of as many as six tip-to-tip transfers needed from a 10 mL PFS, for example, to a 60 mL conventional syringe likely more than offsets the advantage of not having to draw the product from a vial. Indeed, a subsequent 2022 Canadian survey that included patients with 20 mL PFS has shown overwhelming preference for PFS over vials. Furthermore, the additional step in the infusion process may also affect the training experience of these patients, as it would necessitate extra instruction for the tip-to-tip transfer. Indeed, the survey found both the number and length of training sessions were significantly higher for the PFS cohort compared with the vial cohort in the pump subgroup. In contrast, and consistent with this explanation, the number of training sessions was lower in the PFS cohort compared with the vial cohort in the manual push subgroup. It will be important to investigate whether patient perspectives have changed following the subsequent availability of 20 mL PFS in Canada in June 2021, and whether further improvements occur following the recent availability of the even larger 50 mL PFS [41]. Other unknown factors may also have played a role in the greater training time associated with PFS compared with vials.

Third, training patients for self-infusion with SCIg is documented to play an important role in patient reported infusion efficiency and satisfaction [59], and given the global coronavirus disease 2019 (COVID-19) pandemic coincided with certain centers in Canada training patients exclusively with PFS, the sub-optimal shift from in-person to virtual training delivered during the COVID-19 pandemic, although impacting all SCIg patients, is likely to have done so to a greater extent for PFS patients.

Finally, switches to PFS were found to be predominantly driven by physician recommendations and may have reflected some selectivity for those who were intrinsically challenged in terms of dexterity or other functional issues, which may have independently contributed to lower satisfaction [63, 64]. Consistent with this notion, our survey findings suggest that self-reported physical function (measured by PROMIS GPH-2) by PFS users was significantly poorer compared with vial users in the manual push subgroup. In this regard, it is notable that over 90% of patients reported improved outcomes following their switch, with about two thirds of switches being accounted for by a transition from vials to PFS. Further, among those who switched, a numerically higher proportion of those in the PFS cohort reported better outcomes, and in the case of *mental health,* a significantly higher proportion did so, after switching to PFS. These findings together suggest that choice of infusion methods should be tailored to a patient’s needs and lifestyle to optimize outcomes [60–62, 65].

Over 70% of respondents in this survey stated their QoL would improve or substantially improve if their medication could be delivered to their home. There is growing evidence across a wide range of chronic conditions to suggest that mail order pharmacy use is correlated with greater convenience, better medication adherence, improved health outcomes, and decreased healthcare utilization and costs [66, 67]. During the COVID-19 pandemic, patients who were identified as being extremely clinically vulnerable were encouraged to isolate at home, where possible, in many countries to reduce their risk of infection [68, 69]. Home-delivery of SCIg can therefore provide patients with immunodeficiencies access to their treatment without having to travel to a pharmacy or clinic, thereby mitigating the risk of exposure to infections, such as COVID-19.

We acknowledge some limitations are inherent with patient-reported surveys and can result in potentially difficult interpretation. Responses could not be independently verified with patients’ physicians, so the results rely on accurate patient recall and understanding of the survey questions. Missing data points could also impart potential bias. However, the missing data observed in this survey is comparable to a previous study [59]. The survey was also limited to patients who were affiliated with Canadian organizations (i.e., Canadian Immunodeficiencies Patient Organization [CIPO] and APIQ, who are predominantly established in Québec) and generalization of IgRT experiences to wider populations should be made with caution. Despite these potential limitations, our findings on variations of patient-reported treatment satisfaction across SCIg packaging should aid evidence-based decision making and hopefully help to improve patient outcomes.

## Conclusions

In this survey of patients with PID and SID, the SCIg dose delivered via PFS packaging was observed to be significantly lower than that delivered via vials. In addition, patients using PFS reported their pre-infusion preparation times to be significantly quicker than patients using vials. Treatment satisfaction was similar between vial and PFS users in terms of scores on the *effectiveness* and *convenience* domains of the TSQM. Vial users were associated with a greater score on the *global satisfaction* domain of the TSQM compared with the cohort using available small PFS sizes in this survey. Compared with vial users, a higher proportion of patients with shorter experience on PFS and IgRT overall, as well as incompatibility of only the small 5 and 10 mL PFS sizes available at the time, less effective SCIg training during COVID-19 when it mostly occurred, and some physician selectivity in allocating PFS to less able patients with more severe health conditions, may account for these findings. Patient-reported symptom state, overall health status, and mental function were similar across all cohorts. These findings potentially improve our understanding of the impact of different SCIg packaging and infusion methods on treatment satisfaction and PROs, enable best practice for SCIg delivery, and thus may help facilitate optimization of the patient experience.

## Methods

### Data source

Using the CIPO-APIQ database, patients with immunodeficiencies in Canada were contacted via email for purposes of completing an incentivized online survey between October 2020 and March 2021. The survey contained 101 questions on IgRT use and respondent perceptions, as stated in [30], including, but not limited to: respondent characteristics, reasons for choosing SCIg packaging and SCIg administration methods, SCIg self-infusion training experiences, self-infusion characteristics, structured PROs, and details of switching between packaging methods. PROs included (i) the TSQM-9 [70], (ii) PASS [71], to measure symptom status, (iii) GHP [72], to measure overall health related QoL, and (iv) the PROMIS [73], two-item GPH-2 and two-item GMH-2 scales, respectively [74].

### Study exclusion criteria and study cohorts

Respondents were excluded using the following criteria: <18 years old or failure to indicate age, were not currently receiving SCIg incomplete or incongruent responses (i.e., those with incompatible responses such as selecting currently receiving SCIg but citing an IVIg product), failing to indicate their current SCIg packaging and/or SCIg administration method, and indicating they use a mixture of SCIg packaging methods. Respondents were stratified by their current SCIg packaging method into a vial or PFS cohort (Figure 2). The SCIg cohorts were further stratified into subgroups by their SCIg administration method (respondents who infused using a pump or manual push) (Figure 2).

### Outcomes

*Treatment satisfaction* was the primary concept of interest that was expected to be amenable to differences in methods of SCIg packaging. It was assessed using a modified version of the TSQM-9 [70], which measured patients’ satisfaction with medication. In this survey, the instructions to the TSQM-9 asked respondents to focus on the infusion process in their responses, but the wording of the items (questions) themselves was not modified. The TSQM-9 was scored on a verbal rating scale anchored from one to five or seven depending on the question (1–5, where 1 = extremely poor experience/perception and 5 = extremely satisfied experience/perception; 1–7, where 1 = extremely poor experience/perception and 7 = extremely satisfied experience/perception). Raw scores were transformed for each TSQM-9 domain to a 0–100 scale from worst to best.

Patient symptoms, overall well-being and physical and mental function were secondary concepts of interest. Patient *symptom status* was measured using PASS, a single-item, dichotomous measure of patient acceptable symptom state based on a single question, “Considering all the different ways your disease is affecting you, if you would stay in this state for the next months, do you consider that your current state is satisfactory?” [71]. Patients could respond in the affirmative (yes) or in the negative (no).

Overall well-being was encapsulated in the concept of *perceived health status*, measured in terms of the single-item GHP question, “Would you describe your current health status as excellent, very good, good, fair, poor, or very poor?” and 6-point Likert scale scoring (1 = excellent, 2 = very good, 3 = good, 4 = fair, 5 = poor, 6 = very poor) [72]. Responses were dichotomized by combining the excellent, very good, good, and fair categories vs. poor, or very poor for ease of interpretation.

Patient physical function and mental health were assessed using the PROMIS GPH-2 and GMH-2, respectively [74]. Summed scores on each were transformed to corresponding PROMIS T-scores using previously published concordance tables [74].

### Statistical analysis

Categorical outcomes were analyzed using chi-square tests, or Fisher’s exact test when the number in some categories was small. Continuous variables were analyzed using Analysis of Variance (ANOVA) post-hoc tests or unpaired t-tests if found to be normally distributed, or Mann-Whitney tests if otherwise. For the analysis of outcomes (training characteristics, dosing and infusion characteristics, and PROs) a regression analysis was performed to identify any significant (*p*<0.1) interaction between SCIg packaging method and SCIg administration method for a given outcome. Where the interaction was not significant, one analysis for both subgroups was performed, whereas each subgroup was examined separately when the interaction was significant. Outcomes measured on a continuous scale were analyzed using linear regression. Variables with highly positively skewed distributions were analyzed on the log scale in order to meet the analysis assumptions. Binary categorical outcomes were analyzed using binary logistic regression, whilst ordinal categorical outcomes were analyzed using ordinal logistic regression.

### Supplementary Information

**Additional file 1. **Summary of respondent characteristics of the vial and PFS cohorts in the pump subgroup.

**Additional file 2. **Summary of respondent characteristics of the vial and PFS cohorts in the manual push subgroup.

**Additional file 3. **Training characteristics of the vial and PFS packaging cohorts.

**Additional file 4. **Training characteristics of the vial and PFS packaging cohorts in the manual push subgroup.

## Data Availability

The data that was used for this study was obtained from an incentivized survey [30] which was shared with patients whose details are held on the CIPO-APIQ database of patients with immunodeficiencies in Canada. The survey responses are available from the corresponding author upon reasonable request.

## Abbreviations

ANOVA: Analysis of variance
APIQ: Association des Patients Immunodéficients du Québec
CIPO: Canadian Immunodeficiencies Patient Organization
COVID-19: Coronavirus disease 2019
GHP: General health perception
HSCT: Hematopoietic stem cell transplantation
Ig: Immunoglobulin
IgG: Immunoglobulin G
IgRT: Immunoglobulin replacement therapy
IQR: Interquartile range
IVIg: Intravenous immunoglobulin
PASS: Patient acceptable symptom state
PIDs: Primary immunodeficiency diseases
PROMIS: Patient-reported outcomes measurement information system
PROs: Patient related outcomes
SCIg: Subcutaneous immunoglobulin
SF-36: Short-form 36
SIDs: Secondary immunodeficiency diseases
TSQM: Treatment satisfaction questionnaire for medication
QoL: Quality of life
