# Supplementary material for: Subcutaneous immunoglobulin replacement therapy in patients with immunodeficiencies – impact of drug packaging and administration method on patient reported outcomes
Source: BMC Immunol. 2024 Feb 20;25:18. doi: 10.1186/s12865-024-00608-0 (PMC10880328; doi:10.1186/s12865-024-00608-0)
Supplement: Supplementary file 1 — Additional file 1. Summary of respondent characteristics of the vial and PFS cohorts in the pump subgroup. [file 12865_2024_608_MOESM1_ESM.docx]

| **Respondent characteristics  (Pump)** | | **Vial cohort (A)** | | **PFS cohort (B)** | | **p values** |
| --- | --- | --- | --- | --- | --- | --- |
|  |  | **Summary** | **n** | **Summary** | **n** | **A vs. B** |
| Age (years), median [IQR] | | 54 [46, 64] | 38 | 62 [49, 67] | 38 | 0.08 |
| Age at diagnosis (years), median [IQR] | | 47 [31, 54] | 38 | 42 [26, 62] | 38 | 0.36 |
| Gender, n (%) | Female  Male | 26 (68.4%)  12 (31.6%) | 38 | 29 (76.3%)  9 (23.7%) | 38 | 0.44 |
| Weight (kg) | | 77.8 ± 16.9 | 33 | 77.8 ± 21.4 | 35 | 0.99 |
| Underlying condition, n (%) | CVID  IgG Sub  DGS  SID  Other^*^ | 18 (58.1%)  7 (22.6%)  1 (3.2%)  3 (9.7%)  2 (6.5%) | 31 | 15 (51.7%)  3 (10.3%)  3 (10.3%)  5 (17.2%)  3 (10.3%) | 29 | 0.51 |
| Years since diagnosis, n (%) | < 2 years  2–9 years  ≥ 10 years | 3 (7.9%)  21 (55.3%)  14 (36.8%) | 38 | 3 (7.9%)  18 (47.4%)  17 (44.7%) | 38 | 0.71 |
| Time on IgG, n (%) | < 1 year  1–2 years  2–3 years  4–6 years  ≥ 6 years | 2 (5.3%)  4 (10.5%)  3 (7.9%)  13 (34.2%)  16 (42.1%) | 38 | 4 (10.5%)  7 (18.4%)  9 (23.7%)  3 (7.9%)  15 (39.5%) | 38 | 0.17 |
| Current treatment experience, n (%) | < 2 years  2–9 years  ≥ 10 years | 4 (10.5%)  28 (73.7%)  6 (15.8%) | 38 | 9 (23.7%)  18 (47.4%)  11 (29.0%) | 38 | 0.06 |
| Antibiotics before IgG, n (%) | No  Yes | 24 (72.7%)  9 (27.3%) | 33 | 24 (75.0%)  8 (25.0%) | 32 | 0.84 |
| Antibiotics since starting IgG, n (%) | No  Yes | 12 (31.6%)  26 (68.4%) | 38 | 16 (43.2%)  21 (56.8%) | 37 | 0.30 |

**Additional file 1** Summary of respondent characteristics of the vial and PFS cohorts in the pump subgroup.

Data were compared using Mann‑Whitney test, unpaired t‑test, or chi-square test. Significant p-values are highlighted in bold. ^*^Other indications are: X-linked agammaglobulinemia (vial, n=2; PFS, n=0), severe combined immunodeficiency (vial, n=0; PFS, n=1), specific antibody deficiency (vial, n=0; PFS, n=1), hypogammaglobulinemia (vial, n=0; PFS, n=0), Waldenstrom macroglobulinemia (vial, n=0; PFS, n=1). CVID, common variable immune deficiency; DGS, DiGeorge syndrome; GHP, general health perception; GMH-2, global mental health 2; GPH-2, global physical health 2; IgG, immunoglobulin; IgG Sub, immunoglobulin subclass deficiency; IQR, interquartile range; kg, kilogram; PFS, pre-filled syringes; SCIg, subcutaneous immunoglobulin; SD, standard deviation; SID, secondary immunodeficiencies.
