# Supplementary material for: Subcutaneous immunoglobulin replacement therapy in patients with immunodeficiencies – impact of drug packaging and administration method on patient reported outcomes
Source: BMC Immunol. 2024 Feb 20;25:18. doi: 10.1186/s12865-024-00608-0 (PMC10880328; doi:10.1186/s12865-024-00608-0)
Supplement: Supplementary file 2 — Additional file 2. Summary of respondent characteristics of the vial and PFS cohorts in the manual push subgroup. [file 12865_2024_608_MOESM2_ESM.docx]

| **Respondent characteristics (Manual push)** | | **Vial cohort (A)** | | **PFS cohort (B)** | | **p values** |
| --- | --- | --- | --- | --- | --- | --- |
|  |  | **Summary** | **n** | **Summary** | **n** | **A vs. B** |
| Age (years), median [IQR] | | 60 [49, 68] | 28 | 59 [46, 66] | 82 | 0.84 |
| Age at diagnosis (years), median [IQR] | | 45 [20, 60] | 27 | 40 [25, 58] | 80 | 0.54 |
| Gender, n (%) | Female  Male | 13 (48.1%)  14 (51.9%) | 27 | 47 (58.0%)  34 (42.0%) | 81 | 0.37 |
| Weight (kg) | | 71.9 ± 17.4 | 25 | 75.2 ± 15.4 | 74 | 0.37 |
| Underlying condition, n (%) | CVID  IgG Sub  DGS  SID  Other^*^ | 7 (31.8%)  6 (27.3%)  1 (4.6%)  6 (27.3%)  2 (9.1%) | 22 | 22 (34.9%)  23 (36.5%)  1 (1.6%)  5 (7.9%)  12 (19.1%) | 63 | 0.14 |
| Years since diagnosis, n (%) | < 2 years  2–9 years  ≥ 10 years | 1 (3.7%)  13 (48.2%)  13 (48.2%) | 27 | 7 (8.8%)  28 (35.0%)  45 (56.3%) | 80 | 0.40 |
| Time on IgG, n (%) | < 1 year  1–2 years  2–3 years  4–6 years  ≥ 6 years | 1 (3.6%)  3 (10.7%)  1 (3.6%)  8 (28.6%)  15 (53.6%) | 28 | 1 (1.2%)  14 (17.1%)  8 (9.8%)  15 (18.3%)  44 (53.7%) | 82 | 0.77 |
| Current treatment experience, n (%) | < 2 years  2–9 years  ≥ 10 years | 4 (14.3%)  16 (57.1%)  8 (28.6%) | 28 | 12 (15.0%)  46 (57.4%)  22 (27.5%) | 80 | 0.90 |
| Antibiotics before IgG, n (%) | No  Yes | 20 (83.3%)  4 (16.7%) | 24 | 55 (74.3%)  19 (25.7%) | 74 | 0.42 |
| Antibiotics since starting IgG, n (%) | No  Yes | 12 (44.4%)  15 (55.6%) | 27 | 34 (44.2%)  43 (55.8%) | 77 | 0.58 |

**Additional file 2** Summary of respondent characteristics of the vial and PFS cohorts in the manual push subgroup.

Data were compared using Mann‑Whitney test, unpaired t‑test, or chi-square test. Significant p-values are highlighted in bold. ^*^Other indications are: X-linked agammaglobulinemia (vial, n=1; PFS, n=3), severe combined immunodeficiency (vial, n=0; PFS, n=3), specific antibody deficiency (vial, n=1; PFS, n=2), idiopathic autoimmune hemolytic anemia (vial, n=0; PFS, n=3), autoimmune disease (vial, n=0; PFS, n=3), hypogammaglobulinemia (vial, n=0; PFS, n=1), chronic lymphocytic leukemia (vial, n=0; PFS, n=1). CVID, common variable immune deficiency; DGS, DiGeorge syndrome; GHP, general health perception; GMH-2, global mental health 2; GPH-2, global physical health 2; IgG, immunoglobulin; IgG Sub, immunoglobulin subclass deficiency; IQR, interquartile range; kg, kilogram; PFS, pre-filled syringes; SCIg, subcutaneous immunoglobulin; SD, standard deviation; SID, secondary immunodeficiencies.
