# Supplementary material for: Subcutaneous immunoglobulin replacement therapy in patients with immunodeficiencies – impact of drug packaging and administration method on patient reported outcomes
Source: BMC Immunol. 2024 Feb 20;25:18. doi: 10.1186/s12865-024-00608-0 (PMC10880328; doi:10.1186/s12865-024-00608-0)
Supplement: Supplementary file 3 — Additional file 3. Training characteristics of the vial and PFS packaging cohorts. [file 12865_2024_608_MOESM3_ESM.docx]

| **Training characteristics**  **(All respondents)** | | **Vial cohort (A)** | | **PFS cohort (B)** | | **p values** |
| --- | --- | --- | --- | --- | --- | --- |
|  |  | **Summary** | **n** | **Summary** | **n** | **A vs. B** |
| Number of training sessions, n (%) | 1  2  3  4  5  >5 | 32 (51.6%)  17 (27.4%)  9 (14.5%)  1 (1.6%)  2 (3.2%)  1 (1.6%) | 62 | 53 (49.5%)  32 (29.9%)  11 (10.3%)  6 (5.6%)  0 (0.0%)  5 (4.7%) | 107 | 0.79 |
| Location of training, n (%) | Physician’s office  Home  Hospital  Infusion center  Other* | 2 (3.2%)  24 (38.7%)  32 (51.6%)  3 (4.8%)  1 (1.6%) | 62 | 3 (2.8%)  27 (25.2%)  71 (66.4%)  2 (1.9%)  4 (3.7%) | 107 | 0.21 |
| Length of training session (hours), median [IQR] | | 1.3 [1.0, 2.0] | 60 | 2.0 [1.0, 2.0] | 102 | 0.08 |
| Ease of learning to infuse, n (%) | Very difficult  Difficult  Neither  Easy  Very easy | 2 (3.2%)  3 (4.8%)  11 (17.7%)  21 (33.9%)  25 (40.3%) | 62 | 3 (2.8%)  12 (11.2%)  14 (13.1%)  39 (36.5%)  39 (36.5%) | 107 | 0.28 |
| Satisfaction with training, n (%) | Very dissatisfied  Dissatisfied  Neither  Satisfied  Very satisfied | 0 (0.0%)  1 (1.6%)  0 (0.0%)  12 (19.7%)  48 (78.7%) | 61 | 0 (0.0%)  1 (0.9%)  4 (3.7%)  20 (18.7%)  82 (76.6%) | 107 | 0.70 |
| Concerns during training, n (%) | Drawing drug  Inserting needle  Using pump  Prime tube  Other^†^  No concerns | 9 (15.0%)  24 (40.0%)  3 (5.0%)  3 (5.0%)  0 (0.0%)  21 (35.0%) | 62 | 13 (12.2%)  47 (43.9%)  0 (0.0%)  5 (4.7%)  1 (0.9%)  41 (38.3%) | 107 | 0.29 |

**Additional file 3** Training characteristics of the vial and PFS packaging cohorts.

Data were compared using Mann-Whitney test (ease of SCIg training, number of SCIg training sessions) or Fisher’s exact test (SCIg training location, type of SCIg trainer). Significant p values are in bold. *Other training locations: Institut de recherches cliniques de Montréal (vials, n=0; PFS, n=1); †Other concerns: applying needles to tubing (vials, n=0; PFS, n=1). PFS, pre-filled syringes; SCIg, subcutaneous immunoglobulin.
