# Supplementary material for: Subcutaneous immunoglobulin replacement therapy in patients with immunodeficiencies – impact of drug packaging and administration method on patient reported outcomes
Source: BMC Immunol. 2024 Feb 20;25:18. doi: 10.1186/s12865-024-00608-0 (PMC10880328; doi:10.1186/s12865-024-00608-0)
Supplement: Supplementary file 4 — Additional file 4. Training characteristics of the vial and PFS packaging cohorts in the manual push subgroup. [file 12865_2024_608_MOESM4_ESM.docx]

| **Training characteristics**  **(Manual push)** | | **Vial cohort (A)** | | **PFS cohort (B)** | | **p values** |
| --- | --- | --- | --- | --- | --- | --- |
|  |  | **Summary** | **n** | **Summary** | **n** | **A vs. B** |
| Number of training sessions, n (%) | 1  2  3  4  5  >5 | 11 (42.3%)  7 (26.9%)  4 (15.4%)  1 (3.9%)  2 (7.7%)  1 (3.9%) | 26 | 42 (59.2%)  19 (26.8%)  5 (7.0%)  3 (4.2%)  0 (0.0%)  2 (2.8%) | 71 | 0.07 |
| Location of training, n (%) | Doctors  Home  Hospital  Inf Center  Other* | 1 (3.9%)  8 (30.8%)  16 (61.5%)  1 (3.9%)  0 (0.0%) | 26 | 1 (1.4%)  8 (11.3%)  61 (85.9%)  0 (0.0%)  1 (1.4%) | 71 | **0.02** |
| Length of training session (hours) | | 1.3 [1.0, 2.0] | 26 | 1.3 [1.0, 2.0] | 68 | 0.59 |
| Ease of learning to infuse, n (%) | Very difficult  Difficult  Neither  Easy  Very easy | 0 (0.0%)  0 (0.0%)  6 (23.1%)  9 (34.6%)  11 (42.3%) | 26 | 2 (2.8%)  8 (11.3%)  10 (14.1%)  25 (35.2%)  26 (36.6%) | 71 | 0.40 |
| Satisfaction with training, n (%) | Very dissatisfied  Dissatisfied  Neither  Satisfied  Very satisfied | 0 (0.0%)  1 (4.0%)  0 (0.0%)  3 (12.0%)  21 (84.0%) | 25 | 0 (0.0%)  0 (0.0%)  3 (4.2%)  9 (12.7%)  59 (83.1%) | 71 | 0.93 |
| Concerns during training, n (%) | Drawing drug  Inserting needle  Using pump  Prime tube  Other^†^  No concerns | 1 (3.9%)  12 (46.2%)  0 (0.0%)  2 (7.7%)  0 (0.0%)  11 (42.3%) | 26 | 10 (14.1%)  29 (40.9%)  0 (0.0%)  0 (0.0%)  1 (1.4%)  31 (43.7%) | 71 | 0.13 |

**Additional file 4** Training characteristics of the vial and PFS packaging cohorts in the manual push subgroup.

Data were compared using Mann-Whitney test (ease of SCIg training, number of SCIg training sessions) or Fisher’s exact test (SCIg training location, type of SCIg trainer). Significant p values are in bold. *Other training locations: Institut de recherches cliniques de Montréal (vials, n=1; PFS, n=2) or a local community service center (vials, n=0; PFS, n=1); ^†^Other concerns: tip-to-tip transfer (vials, n=0; PFS, n=0). PFS, pre-filled syringes; SCIg, subcutaneous immunoglobulin.
